# Regulation of estrogen signaling and breast cancer proliferation by an ubiquitin ligase TRIM56

**DOI:** 10.1038/s41389-019-0139-x

**Published:** 2019-04-18

**Authors:** Min Xue, Kai Zhang, Kun Mu, Juntao Xu, Huijie Yang, Yun Liu, Beibei Wang, Zhonghao Wang, Zhongbo Li, Qiong Kong, Xiumin Li, Hui Wang, Jian Zhu, Ting Zhuang

**Affiliations:** 1 Henan Key Laboratory of Immunology and Targeted Therapy, School of Laboratory Medicine, Henan Collaborative Innovation Center of Molecular Diagnosis and Laboratory Medicine, Xinxiang Medical University, 453003 Xinxiang, Henan P.R. China; 2 Department of Breast Surgery, Qilu Hospital of Shandong University, 107 West Wenhua Road, 250012 Jinan, Shandong P.R. China; 3 Department of Pathology, School of Basic Medical Sciences, Shandong University, 250012 Jinan, Shandong P.R. China; 4 Rhil Rivers Technology (Beijing) Ltd, Beijing, P.R. China; 5 Department of Cancer Genomics, LemonData Biotech (Shenzhen), Shenzhen, P.R. China; 6 Department of Pharmacology, School of Basic Medical Sciences, Tianjin Medical University, 22 Qixiangtai Road, Heping District, 300070 Tianjin, P.R. China; 7 School of Stomatology, Xinxiang Medical University, 453003 Xinxiang, Henan P.R. China; 8 School of International Education, Xinxiang Medical University, 453003 Xinxiang, Henan P.R. China; 9 Department of Gastroenterology, The Third Affiliated Hospital of Xinxiang Medical University, 453003 Xinxiang, Henan P.R. China; 10 Department of Molecular Biology, University of Texas Southwestern Medical Center, Dallas, TX 75390 USA

**Keywords:** Breast cancer, Hormone receptors

## Abstract

Breast cancer ranks no. 1 in women cancer worldwide, while 60–70% are estrogen receptor alpha positive. The estrogen selective modulators, such as tamoxifen, become the effective drugs for controlling ER alpha breast cancer progression. However, tamoxifen resistance will develop during long-time treatment and cancer progression. Thus, further understanding of ER alpha signaling becomes necessary for the improvement of breast cancer therapy. Here, we identify TRIM56 as a novel regulatory factor in ER alpha signaling. TRIM56 expression is positively correlated with ER alpha and PR in breast cancer samples and is related to poor prognosis in endocrine therapy patients. TRIM56 depletion significantly decreases ER alpha signaling activity and ER-alpha-positive breast cancer proliferation in vitro and in vivo. TRIM56 associates with AF1 domain of ER alpha via its WD40 domain in the cytoplasm. TRIM56 prolongs ER alpha protein stability, possibly through targeting ER alpha K63-linked ubiquitination. In conclusion, our study reveals an interesting posttranslational mechanism between TRIM56 and ER alpha in breast cancer progression. Targeting TRIM56 could be a promising approach for ER-alpha-positive breast cancer.

## Introduction

Breast cancer consists of several subtypes with different criteria, such as pathological type, tumor size, lymph nodes and molecular subtype classification^1^. The most significant advance in recent 20 years is the receptor status classification, which is based on estrogen receptor (ER), progesterone receptor (PR), and human growth factor receptor-2 (HER2) positivity^2^. Among these subgroups, 2/3 breast cancer cases are ER alpha positive, which could be controlled by ER alpha modulators, such as tamoxifen^3^. However, the development of tamoxifen resistance is common, making it an important clinical issue in breast cancer therapy. Besides mutations (Y537S and D538G) in ER alpha AF2 domain, there are a few possible or confirmed mechanisms for endocrine resistance in breast cancer. Some could be functional due to crosstalk between estrogen signaling and other oncogenic pathways, including EGFR, HER2, and NFκB^4,5^. The others could associate with estrogen signaling modulators. For example, ER alpha protein is subject to various kinds of modulations, including phosphorylation, ubiquitination, acetylation and so on, which links to endocrine resistance^6–8^.

The human ER alpha gene was firstly cloned from MCF-7 cells 30 years ago^9^. As a nuclear receptor superfamily member, ER alpha has distinct domains: AF1 domain (Activator Function-1 domain), DBD domain (DNA binding domain), and AF2 domain (Activator Function-2 domain)^10^. With estrogen stimulation, ER alpha translocates into the nucleus and regulates a specific set of gene expression via direct interaction with *cis*-regulatory elements named estrogen-response elements (ERE)^11^. ER alpha has a critical role in breast cancer initiation and proliferation. ER alpha promotes oncogenic protein expression, such as Cyclin D1 and c-Myc, while it inhibits the level of cell cycle inhibitors, including P21 ^12,13^. Since ER alpha signaling is recognized as the driver pathway in the majority of breast cancer patients, the understanding of dysregulation of ER alpha signaling is of utmost importance. Recent studies have shown that several kinds of posttranslational modifications involved in ER alpha stability could contribute to amplified ER alpha signaling and tamoxifen resistance^14,15^. Several E3 ligases are shown to promote ER alpha signaling through stabilizing ER alpha protein, such as RNF31, SHAPRIN, and RNF8 ^7,16,17^.

In our study, we identify the E3 ubiquitin ligase TRIM56 as the novel modulator of ER alpha signaling in breast cancer. TRIM56 is composed of 755 amino acids, while RING domain at its N-terminal is regarded as ubiquitin catalytic domain^18^. Further studies showed that TRIM56 promotes STING K63-linked ubiquitination, which is a process required for antivirus response^18–20^. However, the function of TRIM56 in human malignancies is not clear. Our study reveals a novel modulation of TRIM56 in controlling ER alpha ubiquitination and stability, which subsequently modulates ER alpha target gene expression and ER-alpha-positive breast cancer progression.

## Results

### TRIM56 relates to poor endocrine treatment outcome and correlates with ER alpha and PR protein levels in human breast cancer samples

**Fig. 1:**
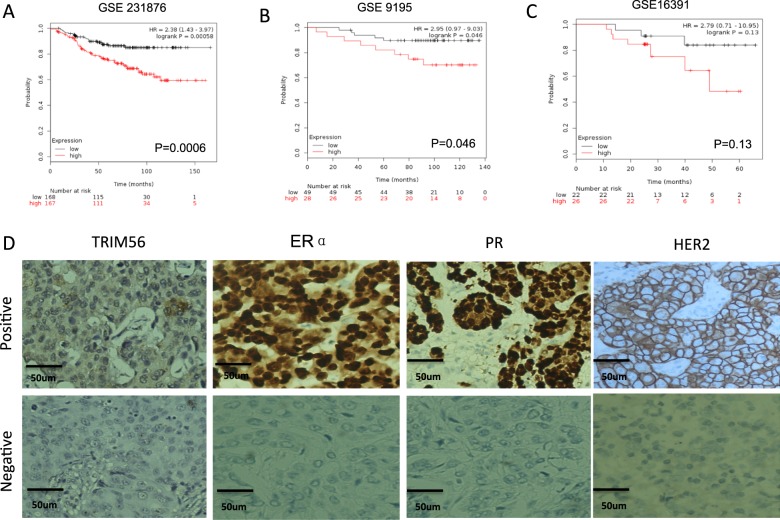
TRIM56 relates to poor endocrine treatment outcome and correlates with ER alpha and PR protein levels in human breast cancer samples. **A–C** TRIM56 mRNA level correlates with poor endocrine treatment outcome in breast cancer patients. Each panel is derived from independent clinical cohorts. These clinical data are acquired from KMPLOT database (http://kmplot.com/analysis/). **D** Examples of positive/negative TRIM56, ERα, PR and HER2 staining in breast tumor samples were shown by ×100 magnification. The statistical data of each protein marker are shown in Table 1

Through the analysis of publicly available clinical breast cancer database (http://kmplot.com/analysis/), we observed that TRIM56 expression level correlates with poor endocrine treatment outcome (Fig. 1A–C), but correlates with favorable survival time in triple-negative breast cancer patients (Supplementary Fig. 1A). In order to analyze the correlation between TRIM56 expression and breast cancer subtype markers, 141 breast tumor tissues were collected and immunohistochemistry (IHC) was applied to examine the protein levels of TRIM56, ER alpha, progesterone receptor (PR), and human epidermal growth factor receptor-2 (HER2) (Fig. 1D). The pathological character and lymph node status data were also collected. The TRIM56 antibody (HPA024358, Sigma-Aldrich) was selected according to the recommendation from ProteinAtlas (https://www.proteinatlas.org/ENSG00000169871-TRIM56/antibody). The IHC results showed that TRIM56 is mainly localized in the cytosol in human breast tumor tissues. The TRIM56 staining was positively correlated with ER alpha and PR in the clinical samples (Table 1). Besides, TRIM56 positivity was also correlated with high pathological grade (Table 1).

**Table 1:** Statistics of clinical features and molecular characteristics

Clinical features and molecular characteristics (cases)	TRIM56			
		+	−	*P* value
ERα	+	59	27	**0.035**
	−	28	27	
PR	+	44	18	**0.04**
	−	43	36	
HER2	+	24	20	0.24
	−	63	34	
Lymph node metastasis	+	44	26	0.78
	−	43	28	
Pathological grade	Low	3	0	**0.04**
	Medium	51	23	
	High	33	31	

Bold text indicates a statistically significant difference with a *p*-value less than 0.05

### TRIM56 depletion inhibits ER-alpha-positive breast cancer cell proliferation in vitro and in vivo

**Fig. 2:**
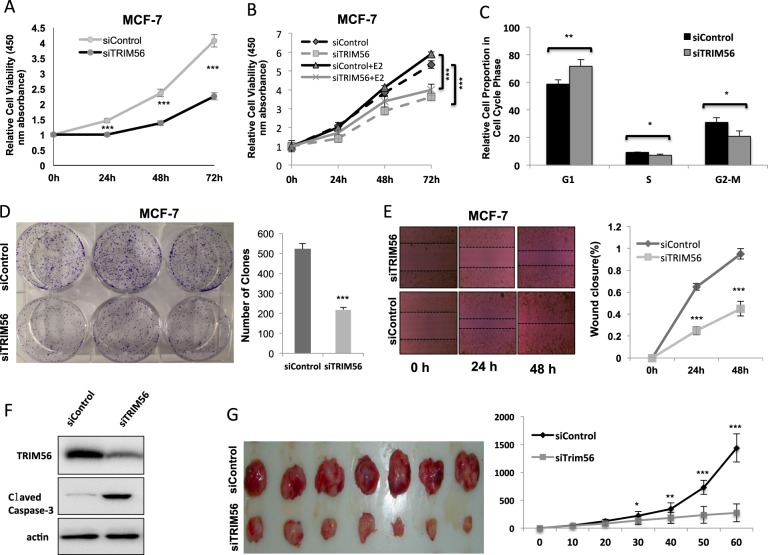
TRIM56 depletion inhibits ER-alpha-positive breast cancer cell proliferation in vitro and in vivo. **A** TRIM56 depletion inhibits the cell proliferation in breast cancer cells. MCF-7 cells were transfected with 50 nM TRIM56 siRNA (mix of #1 and #2) or 50 nM control siRNA. After 24 h, the WST assay was used to determine the cellular metabolic activity at indicated time points after transfection. Experiments were done in triplicates. *P < 0.05; **P < 0.01; ***P < 0.001 for cell growth comparison. **B** TRIM56 depletion inhibits estrogen-driven cell proliferation in breast cancer cells. MCF-7 cells were transfected with 50 nM TRIM56 siRNA (mix of #1 and #2) or 50 nM control siRNA. After 24 h, cells were treated with vehicle or 10 nM estradiol. The WST assay was used to determine the cellular metabolic activity at indicated time points after transfection. Experiments were done in triplicates. *P < 0.05; **P < 0.01; ***P < 0.001 for cell growth comparison. **C** TRIM56 depletion significantly induces G1 cell cycle arrest in breast cancer cells. MCF-7 cells were transfected with 50 nM TRIM56 siRNA (mix of #1 and #2) or 50 nM control siRNA. After 48 h, cells were harvested and fixed by 70% ethanol. The cell cycle phase was anaylzed by PI staining. Experiments were done in triplicates. *P < 0.05; **P < 0.01; ***P < 0.001 for cell growth comparison. **D** Clone formation assay of MCF-7 cells transfected with indicated 50 nM TRIM56 siRNA (mix of #1 and #2) or 50 nM control siRNA. Quantification of clone formation is shown at the indicated time points. Data are presented as ±SD. **P < 0.01, ***P < 0.001 (Student’s *t* test). **E** Wound-healing assay of MCF-7 were transfected with indicated 50 nM TRIM56 siRNA (mix of #1 and #2) or 50 nM control siRNA. Quantification of wound closure at the indicated time points. Data are presented as ±SD. **P < 0.01, ***P < 0.001 (Student’s *t* test). **F** TRIM56 depletion promotes apoptotic signaling in breast cancer cells. T47D cells were transfected with indicated 50 nM TRIM56 siRNA (mix of #1 and #2) or 50 nM control siRNA. After 24 h, cells were harvested for western blot analysis. The cleaved caspase-3 was detected to indicate apoptosis signaling activity. **G** TRIM56 depletion inhibits the cell proliferation in breast cancer cells in vivo. MCF-7 cells were stably transfected with lentivirus carrying a scrambled shRNA or TRIM56 shRNA. Female NOD scid gamma (NSG) mice were estrogen-supplemented by implantation of slow-release 17β-estradiol pellets (0.72 mg/90-d release; Innovative Research of America) 1 day before MCF-7 tumor cell injection into the mammary fat pad (2 × 10^6^ MCF-7 cells suspended in 100 μl Matrigel solution). MCF-7 tumor xenografts were measured every 10 days and the tumor volume was calculated by length × width^2^ /2. The mice were killed 2 months after transplant. The tumor growth curve and a photograph were shown

In order to investigate the potential role of TRIM56 in breast cancer cells, TRIM56 was depleted in MCF-7 and T47D cells. TRIM56 depletion decreased cell proliferation in both MCF-7 and T47D cells (Fig. 2A and Supplementary Fig. 1B), but had no effect on the triple-negative breast cancer cell line (Supplementary Fig. 1C). TRIM56 depletion inhibited cell proliferation in both vehicle and E2-treated conditions (Fig. 2B). Besides, TRIM56 overexpression could further facilitate both MCF-7 cell proliferation and normal epithelial breast cancer cell proliferation (Supplementary Fig. 1D–E). FACS analysis indicated that TRIM56 depletion significantly induced G1 phase arrest (Fig. 2C and Supplementary Fig. 2A). Besides, TRIM56 depletion significantly decreased clone formation capability in MCF-7 and T47D cells (Fig. 2D and Supplementary Fig. 3A–B). Next, we investigated the cancer cell migration capacity in breast cancer cells. Wound-healing assay showed that TRIM56 depletion significantly decelerates cell migration capacity in both MCF-7 and T47D cells (Fig. 2E and Supplementary Fig. 3C–D). Since MCF-7 is deficient in apoptotic signaling, due to a lack of caspase-3, we utilized T47D to observe TRIM56 effect in apoptosis. Our data showed that TRIM56 depletion increased cleaved caspase-3 protein level in T47D cell (Fig. 2F). Then, we further investigated the role of TRIM56 in tumor growth by xenograft mice models. Our data showed that TRIM56 depletion by lentivirus-based shRNA decelerated breast tumor growth (Fig. 2G).

### TRIM56 depletion decreases ER alpha signaling activity in breast cancer cells

**Fig. 3:**
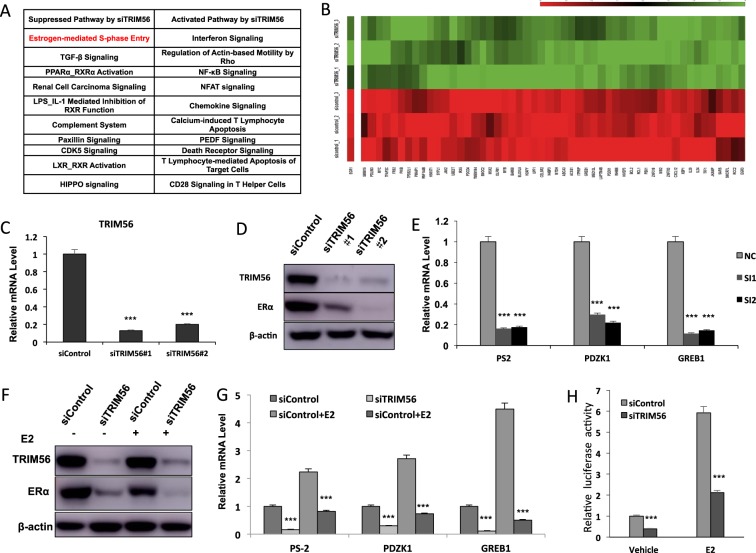
TRIM56 depletion decreases ER alpha signaling activity in breast cancer cells. **A** Top ten signaling pathways significantly decreased/increased by TRIM56 depletion in MCF7 cells. The pathway-enrichment analysis was used by the threshold *P* < 0.001 and fold change >2 to derive regulated genes. TRIM56 was depleted by siRNA (mix of siTRIM56 #1 and siTRIM56 #2) or treated with siControl. After 48 h, the whole mRNA was extracted for RNA sequence analysis. The siControl and siTRIM56 were done in triplicates. **B** The heat-map graph shows the ERα regulating genes, which is significantly inhibited by TRIM56 depletion in MCF-7 cells. The pathway-enrichment analysis was used by the threshold *P* < 0.001 and fold change >2 to derive regulated genes. TRIM56 was depleted by siRNA (mix of siTRIM56 #1 and siTRIM56 #2) or treated with siControl. After 48 h, the whole mRNA was extracted for RNA sequence analysis. The siControl and siTRIM56 were done in triplicates. **C** TRIM56 depletion effect by two different siRNA oligos. MCF-7 cells are transfected with two independent TRIM56 siRNAs or siControl. After 48 h, TRIM56 mRNA levels are determined by QPCR. 36B4 was used as internal control. *P < 0.05; **P < 0.01; ***P < 0.001 for TRIM56 mRNA level comparison. **D** TRIM56 depletion effect on ER alpha protein level by two different siRNA oligos. MCF-7 cells were transfected with two independent TRIM56 siRNAs or siControl. TRIM56 and ER alpha protein levels were determined by the western blot analysis. Actin was used as internal control. **E** TRIM56 depletion decreases ERα target genes using two different siRNA oligos. MCF-7 cells were transfected with siTRIM56 or siControl. After 48 h, total RNA was prepared and the expression of the endogenous ERα target genes, PS2, GREB1, and PDZK1 were determined by qPCR. Shown are the results from three experiments. *P < 0.05; **P < 0.01; ***P < 0.001 for target gene expression comparison. **F** TRIM56 depletion effect on ER alpha protein level. MCF-7 cells were transfected with siTRIM56 or siControl. After 48 h, cells were treated with either ethanol or 10 nM estradiol for 6 h. TRIM56 and ER alpha protein levels were determined by the western blot analysis. Actin was used as internal control. **G** TRIM56 depletion decreases ER alpha target genes. MCF-7 cells were transfected with siTRIM56 or siControl. After 48 h, cells were treated with either ethanol or 10 nM estradiol for 6 h. Total RNA was prepared and the expression of the endogenous ER alpha target genes, PS2, GREB1, and PDZK1 were determined by qPCR. Shown are the results from three experiments. *P < 0.05; **P < 0.01; ***P < 0.001 for target gene expression comparison. **H** TRIM56 depletion affects ERE-luciferase activity in MCF-7 cells. MCF-7 cells were transfected with siTRIM56 or siControl together with ERE luciferase reporter plasmid. Cells were treated with 10 nM estradiol or vehicle. Luciferase activity was measured 48 h after transfection. Shown are the results from three experiments. *P < 0.05; **P < 0.01; ***P < 0.001 for luciferase activity comparison

**Fig. 4:**
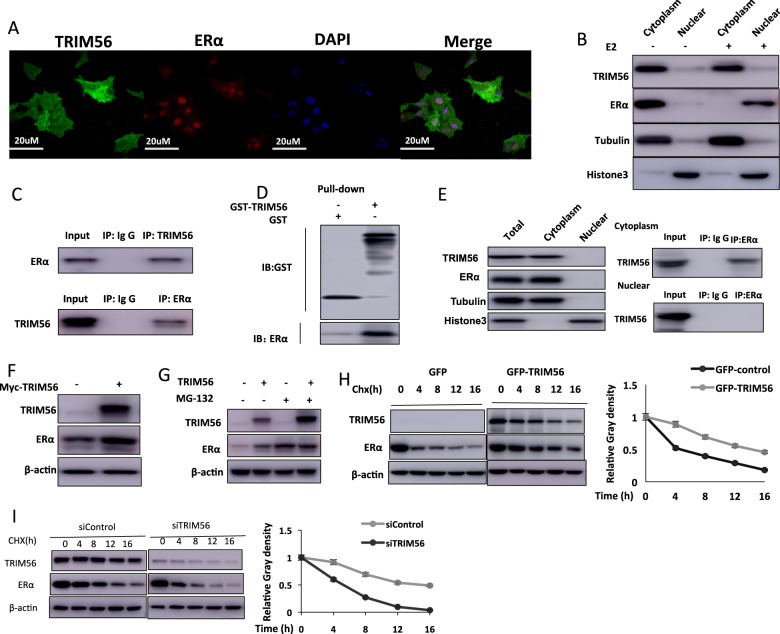
TRIM56 associates with ER alpha and increases its stability. **A** Intracellular localization analysis of TRIM56 and ER alpha by immunofluorescence assay. MCF7 cells were cultured in normal medium before fixation. Intracellular localization of TRIM56 (green) and ER alpha (red) was shown. Nuclei (blue) were stained with 4′,6-diamidino-2-phenylindole (DAPI). **B** TRIM56 protein could not shuttle into the nucleus by estradiol treatment. Cells were subject to estradiol treatment for 30 min. The subcellular protein fractionation kit (Thermo Scientific, 78840) was used for cytoplasm and nuclear separation. Tubulin and Histone-3 were used for cytoplasm and nuclear control. **C** Co-IP assay reveals association between endogenous TRIM56 and ER alpha in MCF7 cells. MCF-7 cells were harvested with RIPA lysis buffer. Co-IP was performed using antibody as indicated. **D** Pulldown assay reveals direct interaction between TRIM56 and ER alpha. ER alpha recombinant protein was incubated with GST-TRIM56 or GST protein. The interacted ER alpha signaling was detected via western blot. **E** TRIM56 is mainly localized in the cytoplasm and associates with ER alpha in the cytosol. The subcellular protein fractionation kit (Thermo Scientific, 78840) was used for cytoplasm and nuclear separation. Tubulin and Histone-3 were used for cytoplasm and nuclear control. Based on the separation, IP was done by TRIM56 antibody in both the cytosol and nuclear lysis. ER alpha antibody was used to detect the interaction in both the cytosol and nuclear. **F** TRIM56 has stabilization effect on ER alpha in HEK293 cells. HEK293 cells were transfected with 2 µg ERα plasmid and 0.5 µg Myc-tag or Myc-TRIM56 plasmids. After 24 h, cell lysates were prepared for western blot analysis. The results are representative for three independent experiments. **G** In the presence of the proteasome inhibitor MG132, the stabilization effect of TRIM56 on ER alpha did not further increase ER alpha protein levels. HEK293 cells were transfected with 2 µg ERα plasmid and 0.5 µg Myc-tag or Myc-TRIM56 plasmids. After 24 h, cells were treated with 10 µM MG132/vehicle for 6 h. Cell lysates were prepared for western blot analysis. The results are representative for three independent experiments. **H** TRIM56 increases ER alpha half-life in HEK293 cells. HEK293 cells were transfected with HA-ERα plasmid and Myc-tag or Myc-TRIM56 plasmids. After 24 h, cells were treated with 100 µM cycloheximide/vehicle for indicated times. Cell lysates were prepared for western blot analysis. The results are representative for three independent experiments. The ER alpha relative density was measured by ImageJ software. **I** TRIM56 depletion decreases ER alpha half-life in MCF-7 cells. MCF-7 cells were transfected with siTRIM56 or siControl. After 24 h, cells were treated with 100 µM cycloheximide/vehicle for indicated times. Cell lysates were prepared for western blot analysis. The results are representative for three independent experiments. The ER alpha relative density was measured by ImageJ software

To approach the function of TRIM56 in breast cancer cells in an unbiased way, we depleted TRIM56 in MCF-7 breast cancer cells for the whole genomic expression analysis. In comparison with siControl cells, TRIM56 depletion was associated with several changes in specific signaling pathways. Pathway analysis showed that TRIM56 depletion decreased TGF-beta signaling, PPAR-RXR signaling, complement signaling, ER alpha signaling and so on. On the other hand, TRIM56 depletion also activated several signaling including interferon signaling and NFκB signaling (Fig. 3A–B). Since ER alpha signaling is predominant in ER-alpha-positive breast cancer cells, we did further analysis on ER alpha target genes expression change by TRIM56 depletion. It was shown that TRIM56 depletion significantly decreased ER alpha target gene expression, including GREB1, PS2, and PDZK1 (Fig. 3B and Supplementary Table 1). Two different individual siRNAs showed that TRIM56 depletion decreased ER alpha protein level and ER alpha target gene expression in MCF-7 cells (Fig. 3C–E). Besides, TRIM56 depletion decreased ER alpha protein level in both E2 and vehicle conditions in MCF7 and T47D cells (Fig. 3F and Supplementary Fig. 4A), while TRIM56 overexpression could increase ER alpha protein level in MCF-7 cells (Supplementary Fig. 4B). Consistent with this, TRIM56 depletion also reduced the expression of endogenous ER alpha target genes such as PS2, GREB1, and PDZK1 in MCF-7 and T47D cells (Fig. 4G and Supplementary Fig. 4C). Depletion of TRIM56 could reduce the expression of endogenous ER alpha target genes such as PS2, GREB1, and PDZK1 in MCF-7 under tamoxifen treatment (Supplementary Fig. 4D). To determine if TRIM56 depletion affected ER alpha transcriptional activity, we measured ER alpha reporter gene activity by TRIM56 depletion. Figure 3H and Supplementary Fig. 4E showed that TRIM56 depletion decreased ER alpha reporter gene activity in the presence and absence of estrogen in MCF-7 and T47D cells, while TRIM56 overexpression could increase ER alpha reporter gene activity (Supplementary Fig. 5A).

### TRIM56 associates with ER alpha and increases its stability

Immunostaining results showed that ER alpha localized mainly in the nucleus under normal medium conditions, while TRIM56 mainly localized in the cytosol (Fig. 4A). Nuclear and cytoplasmic separation assay showed that E2 could promote ER alpha nuclear translocation, while it had little effect on TRIM56 localization (Fig. 4B). Further support for the functional cooperation of TRIM56 and ER alpha was obtained from co-immunoprecipitation (co-IP) of the endogenous proteins from MCF-7 cells. Co-IP showed that TRIM56 could interact with ER alpha (Fig. 4C), but TRIM56 could not associate with ER alpha isoform (ER alpha 36) (Supplementary Fig. 5B). Besides, TRIM56 could enhance the association between ER alpha and NCOA, which functions as an important ER alpha co-activator (Supplementary Fig. 5C). Pulldown assay also detected the direct interaction between ER alpha and TRIM56 (Fig. 4D). Nuclear and cytoplasmic separation-based co-IP showed that TRIM56 as a cytoplasmic protein interacts with ER alpha in the cytoplasm (Fig. 4E). Since it is well known that ER alpha could regulate its own expression in MCF-7 cells, it is difficult to distinguish a direct effect of TRIM56 on ER alpha protein or mRNA levels in the cell line^21^. Thus we performed the protein stability assay in HEK293 cells. ER alpha protein level could be increased, if cotransfected with TRIM56 in HEK293 cells (Fig. 4F). Besides, depletion of endogenous TRIM56 will decrease an exogenously expressed ER alpha Y537S mutant form in the MCF-7 cell (Supplementary Fig. 5D). In the presence of the proteasome inhibitor MG132, the stabilization effect of TRIM56 on ER alpha did not further increase ER alpha protein level (Fig. 4G). Upon inhibition of protein synthesis by cycloheximide, TRIM56 overexpression significantly increased ER alpha protein stability in HEK293 cells (Fig. 4H). Besides, TRIM56 depletion in MCF-7 cells significantly decreased endogenous ER alpha stability (Fig. 4I).

### TRIM56 interacts with ER alpha AF1 domain through its WD40 domain and stabilizes ER alpha possibly through K63-linked ubiquitination

**Fig. 5:**
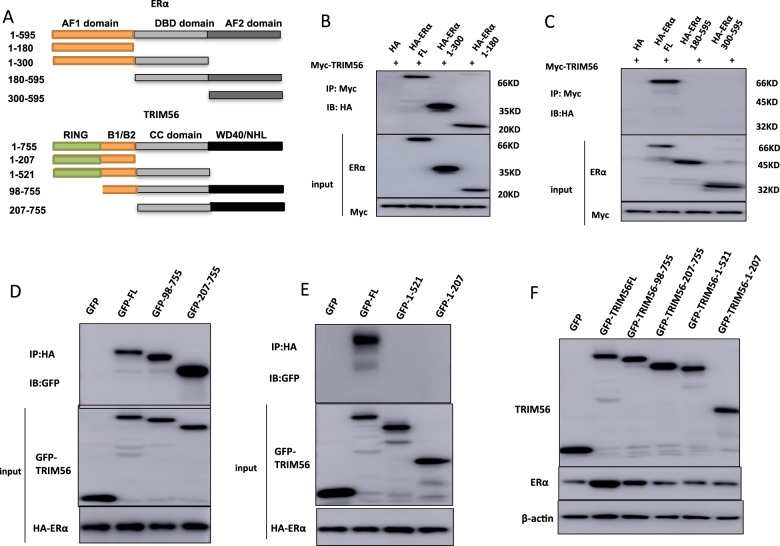
TRIM56 associates with ER alpha AF-1 domain through its WD40 domain. **A** ER alpha domain structure and deletion mutants used in the study (full length, ΔAF1, ΔAF1+ΔDBD, ΔAF2, ΔAF2+ΔDBD). TRIM56 full-length and deletion mutants are used in the study (full length, ΔRING, ΔRING+ΔB1/B2, ΔWD40, ΔWD40+ΔCC domain). **B–C** TRIM56 interacts with ER alpha through its AF1 domain. HEK293 cells were transfected with 2 µg Myc-TRIM56 together with HA-ER alpha full length or mutants (ΔAF1, ΔAF1+ΔDBD, ΔAF2 and ΔAF2+ΔDBD). After 24 h, cells were harvested with NP-40 lysis buffer. Co-IP was performed using Myc antibody. The possible interacted ER alpha domains were detected by HA antibody. **D–E** WD40 domain is required for TRIM56 to interaction with ER alpha. HEK293 cells were transfected with 2 µg HA-ER alpha together with GFP-TRIM56 full length or mutants (ΔRING, ΔRING+ΔB1/B2, ΔWD40, ΔWD40+ΔCC domain). After 24 h, cells were harvested with NP-40 lysis buffer. Co-IP was performed using HA antibody. The possible interacted TRIM56 domains were detected by GFP antibody. **F** The intact TRIM56 domain is necessary for the TRIM56-mediated increase of ER alpha protein level. HEK293 cells were transfected with 2 µg HA-ER alpha together with GFP-TRIM56 full length or mutants (ΔRING, ΔRING+ΔB1/B2, ΔWD40, ΔWD40+ΔCC domain). After 48 h, whole-cell extracts were prepared and the level of ER alpha protein was assayed by western blot analysis

**Fig. 6:**
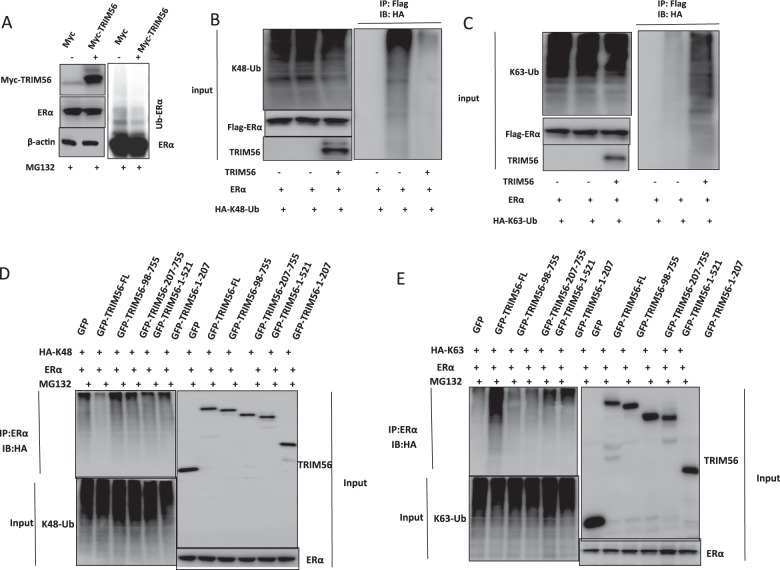
TRIM56 stabilizes ER alpha possibly through K63-linked ubiquitination. **A** TRIM56 prohibits ER alpha polyubiquitination. HEK293 cells were transfected with 2 µg ERα plasmid and 0.5 µg Myc-tag or Myc-TRIM56 plasmids. After 24 h, cells were treated with 10 µM MG132 for 6 h. Cells were directly harvested and western blot analysis using ER alpha antibody was used to detect ubiquitinated ER alpha forms. The predicted molecular weight of polyubiquitinated ERα is indicated. **B** TRIM56 decreases K48-linked polyubiquitination of ER alpha. HEK293 cells were transfected with 2 µg Flag-ER alpha plasmid, 0.5 µg HA-K48 Ubi plasmid and 0.5 µg Myc-TRIM56 plasmids. The cell extracts were immunoprecipitated with HA antibody. The K48-specific polyubiquitinated ER alpha was detected via western blotting analysis. **C** TRIM56 promotes K63-linked polyubiquitination of ER alpha. HEK293 cells were transfected with 2 µg Flag-ER alpha plasmid, 0.5 µg HA-K63 Ubi plasmid and 0.5 µg Myc-TRIM56 plasmids. The cell extracts were immunoprecipitated with HA antibody. The K63-specific polyubiquitinated ER alpha was detected via western blotting analysis. **D** The intact TRIM56 protein is required for its inhibition effect on K48-linked ubiquitination on ER alpha. HEK293 cells were transfected with 2 µg Flag-ER alpha, 0.5 µg HA-K48 Ubi plasmid and 0.5 µg GFP-TRIM56 full length or mutants (ΔRING, ΔRING+ΔB1/B2, ΔWD40, ΔWD40+ΔCC domain). The K48-specific polyubiquitinated ER alpha was detected via western blotting analysis. **E** The intact TRIM56 protein is required for it to facilitate K63-linked ubiquitination on ER alpha. HEK293 cells were transfected with 2 µg Flag-ER alpha, 0.5 µg HA-K63 Ubi plasmid and 0.5 µg GFP-TRIM56 full length or mutants (ΔRING, ΔRING+ΔB1/B2, ΔWD40, ΔWD40+ΔCC domain). The K48-specific polyubiquitinated ER alpha was detected via western blotting analysis

ER alpha has three functional domains: Activation Domain 1 (AF1), DNA binding domain (DBD), and Activation Domain 2 (AF2). TRIM56 has three functional domains: RING domain, B1/B2 domain, and WD40 domain (Fig. 5A). We made these deletion constructs in order to delineate the interaction between ER alpha and TRIM56. The full length of ER alpha or ER alpha deletion constructs (ΔAF1 domain, ΔAF1+ΔDBD domain, ΔAF2 domain, ΔAF2+ΔDBD domain) was expressed together with TRIM56 in HEK293 cells. Co-IP assay indicated that AF1 domain (1–180) was required for ER alpha to interact with TRIM56 (Fig. 5B–C). On the other hand, the full length of TRIM56 or deletion constructs (ΔWD40 domain, ΔRING domain, ΔRING+B1/2 domain and ΔWD40+CC domain) was expressed together with ER alpha in HEK293 cells. Co-IP assay showed that WD40 domain of TRIM56 was necessary for its interaction with ER alpha (Fig. 5D–E). However, by overexpression of TRIM56 full-length or deletion constructs (ΔWD40 domain, ΔRING domain, ΔRING+B1/2 domain, and ΔWD40+CC domain) together with ER alpha into HEK293 cells, we found that intact TRIM56 protein is necessary for its function to stabilize ER alpha (Fig. 5F). The ubiquitin WB assay shows that overexpressed TRIM56 could significantly decrease ER alpha poly-ubiquitination (Fig. 6A). As a ubiquitin ligase, TRIM56 possibly exerts its function via a ubiquitin-based manner. Thus we examined TRIM56 ubiquitination activity on ER alpha protein in three common ubiquitination manners (K48-linked ubiquitination, K63-linked ubiquitination, and monoubiquitination). Ubiquitin-based immunoprecipitation assay showed that TRIM56 could significantly decrease K48-dependent polyubiquitination on ER alpha protein (Fig. 6B), while significantly promoted K63-dependent ubiquitination on ER alpha protein (Fig. 6C). However, TRIM56 did not affect ER alpha monoubiquitination level (Supplementary Fig. 6A). In order to decide which is the functional domain for TRIM56 to modulate ER alpha ubiquitination, TRIM56 full length or deletion constructs (ΔWD40 domain, ΔRING domain, ΔRING+B1/2 domain, and ΔWD40+CC domain) together with ER alpha are transfected into HEK293 cells. Interestingly, only full length of TRIM56 could inhibit K48-linked ER alpha ubiquitination and promote K63-linked ER alpha ubiquitination. This indicates that all the putative domains of TRIM56 are involved in modulating ER alpha ubiquitination (Fig. 6D–E).

## Discussion

**Fig. 7:**
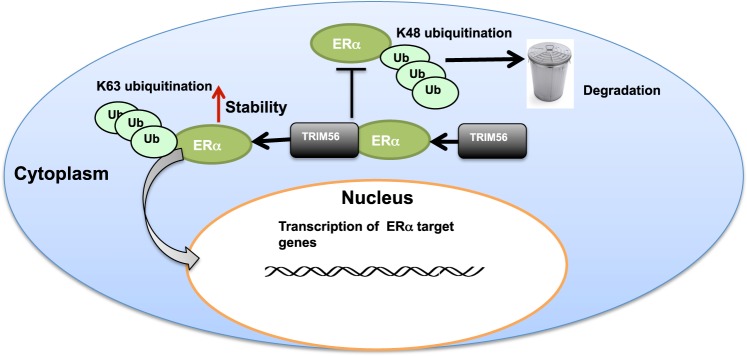
The hypothetical model for TRIM56 regulating ER alpha signaling in breast cancer cells. TRIM56 associates with ER alpha via its WD40 domain and stabilizes ER alpha protein probably via inducing ER alpha K63-linked polyubiquitination and inhibiting ER alpha K48-linked polyubiquitination

Here we report that the RING family ubiquitin ligase TRIM56 associates with and stabilizes ER alpha protein in the cytoplasm in breast cancer cells, which subsequently leads to increased estrogen signaling activity and cell proliferation in vitro and in vivo. Interestingly, TRIM56 is found to promote ER alpha K63-linked ubiquitination, with which a type of ubiquitination is first reported in ER alpha (Fig. 7). On this basis, TRIM 56 inhibition could be a feasible strategy to inhibit cell proliferation in ER-alpha-positive breast cancers.

ER alpha, which belongs to the nuclear receptor superfamily of transcriptional factors, is overexpressed in 60–70% of breast cancers^3^. ER alpha is comprised of three functional domains. The AF-1 domain could transactivate transcription in the absence of ligand binding. The DNA binding domain binds to estrogen response elements in DNA, while the AF2 domain is the ligand-dependent transactivation domain. The AF2 domain could also bind to several coactivators or corepressors of ER alpha^10^. When ER alpha is stimulated with estrogen, the ER alpha protein could translocate into the nucleus and bind to *cis*-regulatory DNA regions of target genes and facilitate gene expression^11^. Since two thirds of breast cancers have ER alpha overexpression, endocrine therapy targeting ER alpha signaling has been proved as a successful strategy for ER-alpha-positive breast cancer patients^3^. Quite a lot of confirmed and hypothetic mechanisms have been reported for endocrine resistance. Besides the low percentage of ER alpha gene amplifications and ER alpha gene mutations in AF-2 domain, endocrine resistance could mainly associate with two categories^22,23^. For example, ER alpha could crosstalk with several other signaling pathways, such as HER2, EGFR and NFκB signaling, which could facilitate cell proliferation and tamoxifen resistance^4,5^. ER alpha interacts with HER2 protein, resulting in the activation of MAPK signaling, while the activation of MAPK pathway could also promote ER alpha phosphorylation and enhance ER alpha signaling activity^24^. In clinics, ER alpha and HER2 crosstalk provide an explanation why ER positive plus HER2 positive patients show a lower tamoxifen efficacy^24^. In addition to crosstalk mechanisms, the modification of ER alpha signaling could affect endocrine therapy efficacy through several mechanisms. For example, ER alpha signaling activity could be modified by posttranslational modifications, such as phosphorylation, ubiquitination, and acetylation. One example is that P300 functions to promote ER alpha acetylation at the hinge domain and subsequently promote ER signaling activity^8^. Another is that ER alpha phosphorylation at certain sites could change ER alpha signaling activity and tamoxifen inhibition efficacy. The phosphorylation at Y537 site changes helix loop conformation and subsequently increases ligand binding/coactivator binding efficiency^25^.

The E3 ubiquitin ligases could be divided into two groups according to the function of ubiquitin catalytic domain: the HECT group and the RING group^26^. There are about 30 different HECT E3 ligases, whose function involves in protein transfer, immune response, and DNA repair process^26^. However, compared with the HECT family of E3 ligase, there are about 700 different RING family E3 ligases, most of which are not well studied^27^. Unlike the classical E3 ubiquitin ligases, recent studies showed that RING family E3 ligases often stabilize their substrates, by which the mechanism is often substrate dependent^28^. Since E3 ubiquitin ligases catalyze the transfer of ubiquitin from E2s to their lysine of substrates, the lysine residues on the ubiquitin vary, which could account for different subsequent biology processes^29^. For example, K48-linked ubiquitin process is always related to proteasome-dependent degradation, while K63-linked ubiquitin or monoubiquitin process could be a change of substrate functions, such as signaling transduction or protein trafficking process^18,30^.

TRIM56 protein was first characterized as a key modulator in regulating innate immune response via interacting with STING and promoting STING K63-linked ubiquitination, which is a process required for the recruitment of TBK1 and induction of IFN-beta^18^. Further studies showed TRIM56 promotes another type of ubiuiqitination to cGAS^31^. Besides, TRIM56 is inducible by virus or IFN-beta and is essential for TLR3 signaling in response to virus infection^19^. However, the detailed role of TRIM56 in cancer progression is still not clear, although some studies showed TRIM56 could suppress malignancy progression in ovary cancer and multiple myeloma^32,33^. In our study, we identify that TRIM56 exerts its stabilization effect on ER alpha protein, which is dependent on both the RING domain at the N-terminal and the WD40 domain at the C-terminal. Although TRIM56 was capable to induce monoubiquitination in other studies, we failed to detect its effect on ER alpha. There are two possible explanations for the stabilization effect on ER alpha. One is that TRIM56 exerts its stabilization function in a K63-dependent manner. Another is that TRIM56 binds to ER alpha and prohibits ER alpha K48-linked ubiquitination and degradation, in which the stabilization effect is not dependent on K63 ubiquitination. However, our data indicate that deletion of RING domain, which is required for E3 ligase catalytic function, will lose ER alpha stabilization effect, although TRIM56 RING domain deletion could still interact with ER alpha. Further ubiquitin immunoprecipitation also showed that loss of the RING domain could not promote K63-linked ubiquitination of ER alpha, and also could not inhibit K48-linked ubiquitination of ER alpha. These might indicate that TRIM56 stabilizes ER alpha possibly through inducing K63-linked ubiquitination. However, It remains to be determined that why K63-linked ubiquitination could increase ER alpha protein stability and subsequently enhanced ER alpha signaling activity. In the study, we examine the role of TRIM56 in ER-alpha-positive breast cancer cells. TRIM56 is shown to interact with ER alpha protein and prolong its stability probably via K63-linked ubiquitination at ER alpha. Since the ER alpha signaling is required for breast cancer proliferation, modulation of ER alpha protein could be an approach to inhibit breast cancer cell progression and restore endocrine resistance. In all, targeting TRIM56 could be a promising therapeutic strategy for breast cancer treatment.

## Materials and methods

### Cell culture

MCF-7, T47D, and HEK293 cells were obtained from American Type Culture Collection (ATCC). MCF-7 and HEK293 were grown in Dulbecco’s modified Eagle’s medium (DMEM) that contains 4.5 g/l glucose and 4 mM l-glutamine (DMEM, 41965, Life Technologies) supplemented with 10% fetal bovine serum (FBS, 10270, Life Technologies). T47D cells were grown in RPMI-1640 (42401, Life Technologies) supplemented with 2 mM l-glutamine (25030, Life Technologies) and 10% FBS. All cell lines were subject to cell line authentication. The cell line authentication via Short Tandem Repeat (STR) was performed via PowerPlex 21 system. The STR data of MCF-7 and T47D cell lines were found consistent with STR data in ATCC.

### Plasmids

The Myc-tag-TRIM56 plasmid was acquired from Origene (RC200891). The TRIM56 delta-RING construct, delta-RING-B1/2-RING construct, delta-WD40 construct, and delta-WD40-CC domain construct were subcloned from the original plasmid. The ER alpha full- and deletion constructs were described in a previous study. The HA-K48 and HA-K63 Ubi plasmids were gifted from Dr. Bo Yang and Jie Wang^34^. The HA-Ub-KO plasmids were described in our previous study. The ERE-TK reporter and renilla plasmids were transfected with Lipofectamin 200 (1662298, Invitrogen)

### RNA extraction and qPCR analysis

RNeasy plus mini kits were used to extract total RNA (Qiagen). Real-time PCR was performed as previously described^35^. 36B4 was used as internal control. Primer sequences for qPCR are provided in Table 2.

**Table 2:** qPCR primers used in this study

Primer sequences for QPCR		
GREB1	Forward	cgt gtg gtg act gga gta gc
	Reverse	acc tct tca aag cgt gtc gt
PS2	Forward	cat cga cgt ccc tcc aga aga g
	Reverse	ctc tgg gac taa tca ccg tgc tg
PDZK1	Forward	gcc agg ctc att cat caa aga
	Reverse	cct cta gcc cag cca agt ca
ERα	Forward	gct acg aag tgg gaa tga tga aag
	Reverse	tct ggc gct tgt gtt tca ac
36B4	Forward	ggc gac ctg gaa gtc caa ct
	Reverse	cca tca gca cca cag cct tc
TRIM56	Forward	tca aga gca agt ggc cca ag
	Reverse	ccg tgg gaa acc atg ctg aa

### Quantification of cell viability

MCF-7 and T47D cells were transfected with siTRIM56 or siControl in 24-well plates. Twenty-four hours after transfection, the cell number was countered and 4000 cells were seeded into 96-well plates. The relative cell viability was measured at indicated time points. Cell numbers were determined using the WST-1 cell proliferation reagent as previously described^35^.

### Xenograft tumor model

MCF-7 cells were infected with shControl virus or shTRIM56 virus (TR30013 and TL300830V, Origene). After 48 h of infection, cells were selected with 1 μg/ml puromycin for 3 days. The female nonobese diabetic-SCID mice were implanted with slow-release 17 beta-estradiol pellets (0.72 mg/90-day, Innovative Research of America). After 24 h, about one million MCF-7 cells together with matrigel solution were injected into the mammary fat pad for each mouse. The tumor sizes were measured every 10 days. After 60 days, the mice were sacrificed and the tumors were weighted and photographed. The experiments were performed under the protocols approved by ethnic committee of Xinxiang Medical University.

### Wound-healing assay

50 nM TRIM56 siRNA or siControl was transfected into MCF-7 and T47D cells. After 24 h, cells were seeded into 12-well plates with 1% FBS. The cells were 100% confluent. The yellow pipette tips were applied for straight scratch. The wound distance was measured at indicated time points and normalized with starting time point. The wound-healing recovery was expressed as: [1 − (Width of the wound at a given time/width of the wound at *t* = 0)] × 100%.

### Clone formation assay

MCF-7 and T47D cells were seeded in six-well plates overnight and treated with 50 nM TRIM56 siRNA or 50 nM siControl. Twenty-four hours posttransfection, the cells were washed with PBS, trypsinized and plated at low density (5000 cell/well in six-well plate). The cells were cultured for 10 days and the medium was refreshed every 2 days. The colonies were stained with crystal violet. The number of the clones in a given area was counted for each condition.

### Flow cytometry analysis

MCF-7 cells were seeded into 10-cm dishes. The cells were transfected with TRIM56 siRNA or siControl. After 24 h, cells were fixed in 70% ethanol for 20 min and incubated with propidium iodide. After that, each group of cells was used to measure the flow fluorescence intensity. The cell cycle phases were determined by relative DNA content. The siTRIM56/siControl groups were done in triplicates.

### Western blotting

Cells were harvested and lysed with RIPA buffer. Proteins were separated by electrophoresis on SDS-polyacrylamide gel electrophoresis (PAGE) and electro-transferred to PVDF membrane. The antibodies used in this study are listed here: Anti-ER alpha (D8H8, 8644, Cell Signaling Technology); Anti-ER alpha (SC-56833, Santa Cruz); Anti-HA (MMS-101R, COVANCE); Anti-myc (9E10, ab32, Abcam); Anti-myc (Ab9106, Abcam); TRIM56 (Ab154862, Abcam); Anti-Flag (Ab49763, Abcam); Anti-GFP (Ab290, Abcam); Anti-cleaved caspase-3 (Ab2302, Abcam). Membranes were then washed with PBS for three times and incubated with secondary antibodies peroxidase-conjugated AffiniPure goat anti-mouse IgG or goat anti-rabbit IgG. Fluorescent signals were visualized with ECL system (Amersham imager 600, USA).

### Luciferase assay

The luciferase activity of estrogen signaling activity was performed using the Dual-Luciferase Reporter kit (Promega, Germany). The ERE luciferase reporter was transfected together with the Renilla plasmid into the cells. Luciferase activity was measured after 24 h.

### Co-immunoprecipitation assay

Immunoprecipitation was performed as described in previous study^36^. The MCF-7 total cell lysis were precleared with rabbit IgG for 2 h and subsequently immunoprecipitated with ER alpha antibody (D8H8, #8644) overnight, while rabbit IgG (Santa Cruz) was used as the negative control. The bounded protein was analyzed by Anti-TRIM56 antibody (AB154862). For the overexpression experiment, HEK293 cells were transfected with 5 μg GFP-TRIM56 (full length or deletion domains) and ER alpha plasmid (full length or deletion domains) in 10 cm dish. Cell lysates were precleared with IgG and subsequently incubated with GFP (AB190) antibody or Flag (Ab49763) antibody, while rabbit IgG was used as the negative control. The bound proteins were analyzed by western blotting.

### Pulldown assay

ERα recombinant protein was acquired from Abcam (Ab82606). GST protein and GST-fusion TRIM56 proteins were acquired from Samgon Biotech (China). The mixture was incubated at 4 °C with rotation for 30 min; the resin was washed twice with PBS containing 30 mM imidazole, followed by washing twice with PBS containing 0.01% Triton X-100. Bound proteins were eluted with elution buffer (50 mM sodium phosphate, 300 mM NaCl, 250 mM imidazole [pH 7.4]) and subjected to SDS-PAGE analysis.

### Protein stability assays

About 10^5^ HEK293 cells were seeded into 24-well plates and transfected with 0.5 μg ER alpha plasmid together with 0.5 μg Myc-TRIM56 or Myc-vector. After 48 h, cells were treated with 100μM cycloheximide (C7698, Sigma) for indicated time points. Samples were subject to western blot for ER alpha degradation. For MCF-7 cells, 10^5^ cells were seeded into 24-well plate and transfected with 50 nM siTRIM56 or siControl. After 24 h, cells were treated with 100 μM cycloheximide (C7698, Sigma) for indicated time points. Samples were subject to western blot for ER alpha degradation.

### Analysis of protein ubiquitination

HEK293 cells were transfected with 2 μg ER alpha plasmid together with 2 μg Myc-TRIM56 or Myc-vector. After 48 h, cells were treated with 10 μM MG132 (474787, Sigma) for 6 h. Cells were directly harvested. The polyubiquitination of ER alpha was detected by direct western blotting analysis against ER alpha.

### Polyubiquitination detection assay

To directly detect the enriched K48-ubiquitinated, K63-ubiqutinated or monoubiquitinated ER alpha from the cell extracts, HEK293 cells were transfected with 0.5 μg K48 Ubi, 4 μg K63 Ubi, or 4 μg Ub-KO plasmid, 2 μg ER alpha together with 0.5 μg Myc-TRIM56 or Myc-vector. After 48 h, total protein was extracted and precleared with 20 μl protein A (Santa Cruz, SC-2001) for 2 h. The supernatant was collected and immunoprecipitated by ER alpha antibody. Western blot with HA antibody was performed to detect K48, K63 polyubiquitinated or monoubiquitinated ER alpha.

### Immunofluorescence assay

MCF-7 cells were fixed with 4% paraformaldehyde in PBS for 10 min, permeabilized with 0.2% Triton X-100 for 5 min, and blocked by 5% BSA in PBS for 1 h. A rabbit anti-TRIM56 polyclonal antibody (Ab154862) and mouse anti-ERα monoclonal antibodies (SC-56833) were used, followed by Alexa Flour 647 (Invitrogen) anti-rabbit antibody and FITC-conjugated anti-mouse antibodies (Jackson ImmunoResearch, West Grove, PA). As negative controls, the samples were incubated with the secondary antibodies without primary antibodies. Images were acquired under conditions fulfilling the Nyquist criterion using Nikon A+ laser scanning confocal system with a ×60 oil NA1.4 objective and pinhole size of 1.0 Airy Unit. The acquired pictures were further processed and assembled using ImageJ.

### Clinical breast tumor samples

One hundred and forty-one formalin-fixed paraffin-embedded breast cancer samples were collected from the Department of Pathology, Shandong Qilu Hospital. All the breast tumors samples were examined by ERα status, PR status, HER2 status by pathological specialists. The pathological grade plus lymph node metastasis status of each sample was also examined by pathological specialists. This study was reviewed and approved by the Ethical Board at the Qilu Hospital of Shandong University with written informed consent from all the patients.

### RNA sequence analysis

The global gene expression analysis (siControl and siTRIM56) was based on RNA sequencing platform from BGI (Beijing Genomic Institute). The RNA sequence data are deposited in the Gene Expression Omnibus (GEO) database (Assessing number: GSE122158). Analysis was performed for differentially expressed genes (*P* < 0.01 and fold change >2) by Ingenuity Pathway Analysis (IPA).

### Statistics

Student’s *t* test, Pearson correlation coefficient, and Cox regression analysis were used for comparisons. A *P* value of <0.05 was considered to be significant.

## Supplementary information

Supplementary figure legends

Supplementary Figures

Supplementary table

## Data Availability

Additional data are available as Supplementary information.
