# Supplementary material for: Regulation of estrogen signaling and breast cancer proliferation by an ubiquitin ligase TRIM56
Source: Oncogenesis. 2019 Apr 18;8(5):30. doi: 10.1038/s41389-019-0139-x (PMC6473003; doi:10.1038/s41389-019-0139-x)
Supplement: Supplementary file 1 — Supplementary figure legends [file 41389_2019_139_MOESM1_ESM.docx]

**Supplementary Figure legends**

**Figure 1A:** TRIM56 expression correlates with good prognosis in triple negative breast cancer patients. The data is generated from KMPLOT public available database (http://kmplot.com/analysis/index.php?p=service&cancer=breast).

**Figure 1B:** TRIM56 depletion inhibits the cell proliferation in breast cancer cells. T47D cells were transfected with 50nM TRIM56 siRNA (mix of #1 and #2) or 50nM control siRNA. After 24 hours, the WST assay was used to determine the cellar metabolic activity at indicated time points after transfection. Experiments were done in triplicates. *P<0.05; ** P<0.01; ***P<0.001 for cell growth comparison.

**Figure 1C:** TRIM56 depletion does not affect cell proliferation in MDAMB231 cells. MDAMB231 cells were transfected with 50nM TRIM56 siRNA (mix of #1 and #2) or 50nM control siRNA. After 24 hours, the WST assay was used to determine the cellar metabolic activity at indicated time points after transfection. Experiments were done in triplicates. *P<0.05; ** P<0.01; ***P<0.001 for cell growth comparison.

**Figure 1D:** TRIM56 overexpression promotes breast cancer cell proliferation in MCF-7 cells. MCF-7 cells were seeded into 6-well plate. After 24 hours, cells were transfected with 1 ug Myc-TRIM56 plasmid or 1ug Myc-tag plasmid. Twenty-four hours post-transfection, cells were seeded into 96-well plate. After 24 hours, the WST assay was used to determine the cellar metabolic activity at indicated time points after transfection. Experiments were done in triplicates. *P<0.05; ** P<0.01; ***P<0.001 for cell growth comparison.

**Figure 1E:** TRIM56 overexpression promotes breast normal epithelial cell proliferation in MCF-10A cells. MCF-10A cells were seeded into 6-well plate. After 24 hours, cells were transfected with 1 ug Myc-TRIM56 plasmid or 1ug Myc-tag plasmid. Twenty-four hours post-transfection, cells were seeded into 96-well plate. After 24 hours, the WST assay was used to determine the cellar metabolic activity at indicated time points after transfection. Experiments were done in triplicates. *P<0.05; ** P<0.01; ***P<0.001 for cell growth comparison.

**Figure 2:** TRIM56 depletion significantly induces G1 cell cycle arrest in breast cancer cells. MCF-7 cells were transfected with 50nM TRIM56 siRNA (mix of #1 and #2) or 50nM control siRNA. After 48 hours, cells were harvested and fixed by 70% ethanol. The cell cycle phase was anaylsis by PI staining. Experiments were done in triplicates. *P<0.05; ** P<0.01; ***P<0.001 for cell growth comparison. The dot plot and histograms of siTRIM56 and siControl were shown.

**Figure 3A and 3B:** Clone formation assay of T47D cells were transfected with indicated 50nM TRIM56 siRNA (mix of #1 and #2) or 50nM control siRNA. Quantification of clone formation is shown at the indicated time points. Data are presented as ± SD. **, P<0.01, ***, P< 0.001 (student’s t-test).

**Figure 3C and 3D:** Wound healing assay of T47D were transfected with indicated 50nM TRIM56 siRNA (mix of #1 and #2) or 50nM control siRNA. Quantification of wound closure at the indicated time points. Data are presented as ± SD. **, P<0.01, ***, P< 0.001 (student’s t-test).

**Figure 4A:** TRIM56 depletion effect on ER alpha protein level. T47D cells were transfected with siTRIM56 or siControl. After 48 h, cells were treated with either ethanol or 10nM estradiol for 6 h. TRIM56 and ER alpha protein levels were determined by Western blot analysis. Actin was used as internal control.

**Figure 4B:** TRIM56 overexpression could increase ER alpha protein level in MCF-7 cells. MCF-7 cells were seeded into 6-well plate. After 24 hours, cells were transfected with 1 ug Myc-TRIM56 plasmid or 1ug Myc-tag plasmid. Twenty-four hours post-transfection, cells were harvested for western blot analysis. TRIM56 and ER alpha protein levels were determined by Western blot analysis. Actin was used as internal control.

**Figure 4C:** TRIM56 depletion decreases ER alpha target genes. T47D cells were transfected with siTRIM56 or siControl. After 48 h, cells were treated with either ethanol or 10nM estradiol for 6 h. Total RNA was prepared and the expression of the endogenous ER alpha target genes, PS2, GREB1, and PDZK1 were determined by qPCR. Shown are the results from three experiments. *P<0.05; ** P<0.01; ***P<0.001 for target gene expression comparison.

**Figure 4C:** TRIM56 depletion decreases ER alpha target genes under tamoxifen-treated condition. MCF-7 cells were transfected with siTRIM56 or siControl. After 48 h, cells were treated with either ethanol or 1 uM tamoxifen for 6 h. Total RNA was prepared and the expression of the endogenous ER alpha target genes, PS2, GREB1, and PDZK1 were determined by qPCR. Shown are the results from three experiments. *P<0.05; ** P<0.01; ***P<0.001 for target gene expression comparison.

**Figure 4D:** TRIM56 depletion affects ERE-luciferase activity in T47D cells. T47D cells were transfected with siTRIM56 or siControl together with ERE luciferase reporter plasmid. Cells were treated with 10 nM estradiol or vehicle. Luciferase activity was measured 48 h after transfection. Shown are the results from three experiments. *P<0.05; ** P<0.01; ***P<0.001 for luciferase activity comparison.

**Figure 5A:** TRIM56 increases ER alpha response element activity in MCF-7 cells. MCF-7 cells were transfected with 0.5 µg Myc-tag or TRIM56 plasmids together with ERE luciferase plasmids. Cells were treated with 10 nM estradiol or vehicle. Luciferase activity was measured 48 h after transfection. Shown are the results from three experiments. *P<0.05; ** P<0.01; ***P<0.001 for luciferase activity comparison.

**Figure 5B:** TRIM56 associates with ER alpha full length but not with ER alpha 36 variant. MDAMB231 cells were transfected with 1 ug ER alpha full-length plasmid. After 24 hours, cells were harvested for immuno-precipitation. CO-IP was performed using TRIM56 antibody. ER alpha full-length and variant were detected by antibody against ER alpha C-terminus.

**Figure 5C:** TRIM56 could facilitate ER alpha-NCOA interaction. HEK293 cells were transfected with 2 µg HA-ER alpha, 2 µg Myc-TRIM56 and 2 µg EGFP-NCOA1 in different combination. After 24 h, cells were harvested with the cytoplasmic protein fractionation kit (Thermo scientific, 78840). CO-IP was performed using HA antibody. The possible interacted NCOA1 were detected by GFP antibody.

**Figure 5D:** TRIM56 depletion could de-stabilize ER alpha Y537S mutant form. MCF-7 cells were transfected with 1 ug ER alpha Y537S mutant plasmid. After 24 hours, cells were transfected with 50 uM siTRIM56 or siControl. Twenty-four hous post-transfection, cells were harvested for western blot analysis. ER alpha Y537S protein levels were determined by Flag antibody. Actin was used as internal control.

**Figure 6A:** TRIM56 does not affect mono-ubiquitination of ER alpha. HEK293 cells were transfected with 2 µg Flag-ER alpha plasmid, 0.5 µg HA-UbKO plasmid and 0.5 µg Myc-TRIM56 plasmids. The cell extracts were immunoprecipitated with HA antibody. The Mono-ubiquitinated ER alpha was detected via western blotting analysis.
