# Supplementary material for: Regulation of estrogen signaling and breast cancer proliferation by an ubiquitin ligase TRIM56
Source: Oncogenesis. 2019 Apr 18;8(5):30. doi: 10.1038/s41389-019-0139-x (PMC6473003; doi:10.1038/s41389-019-0139-x)

Supplementary figure 1

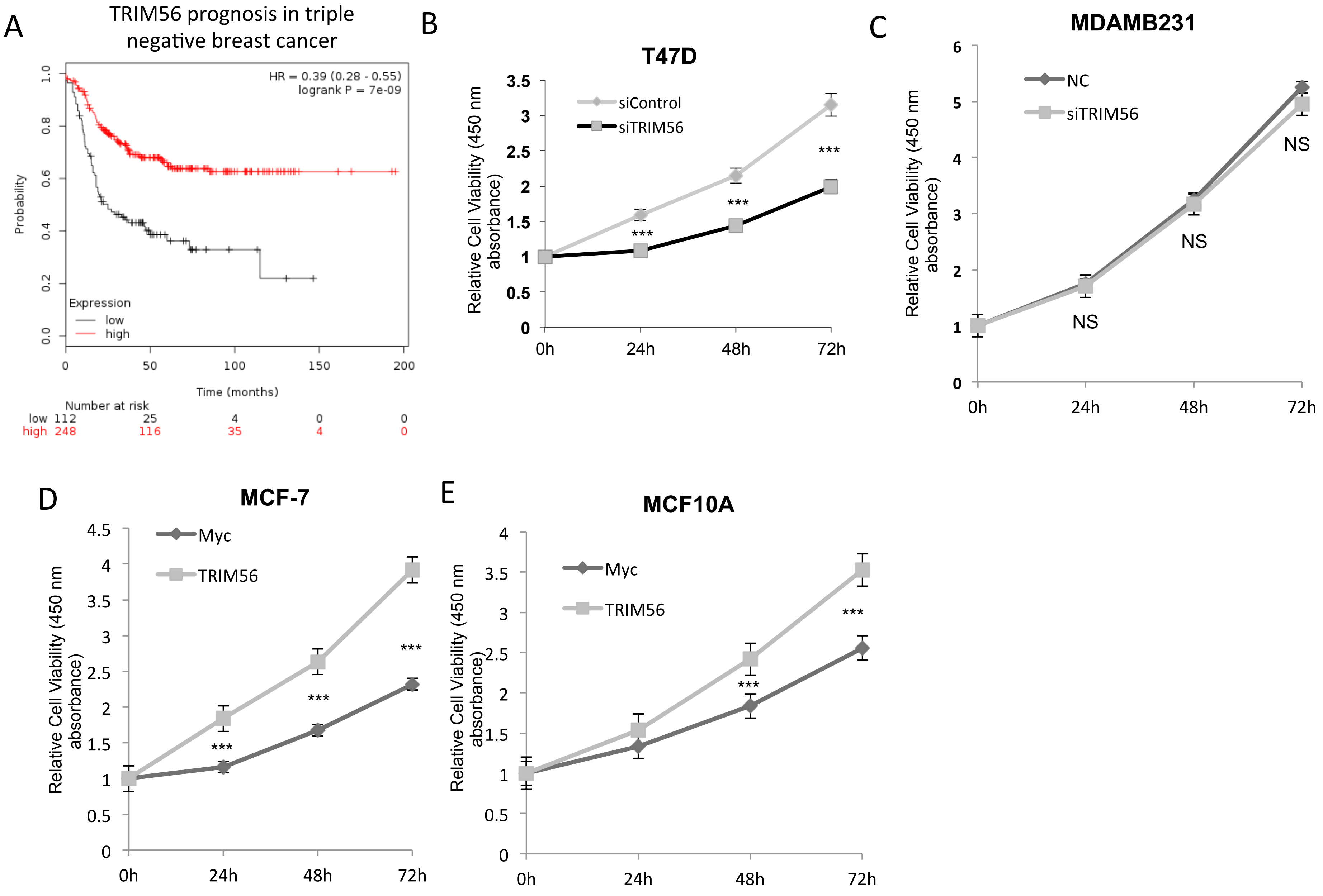

Supplementary figure 2

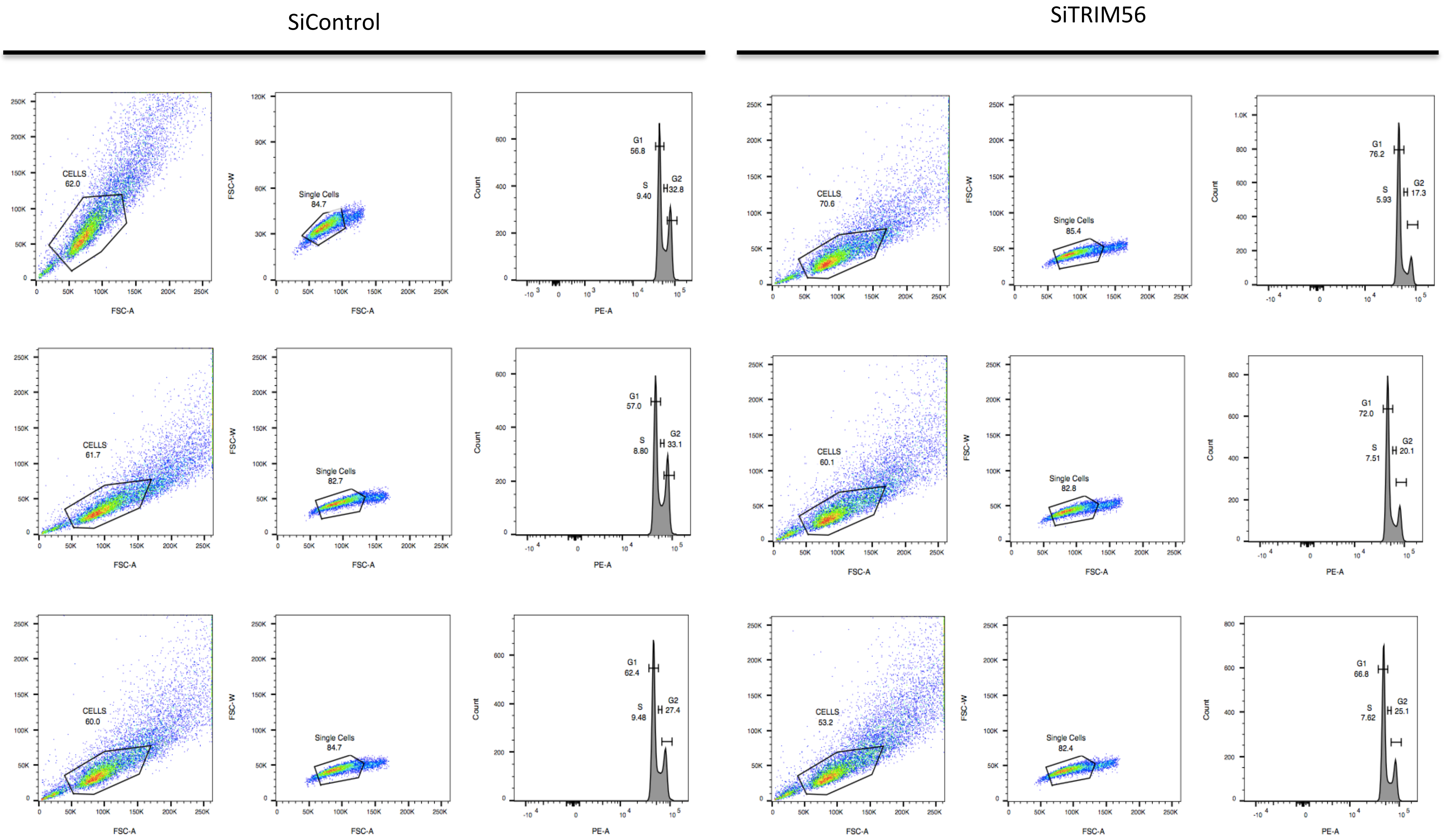

Supplementary figure 3

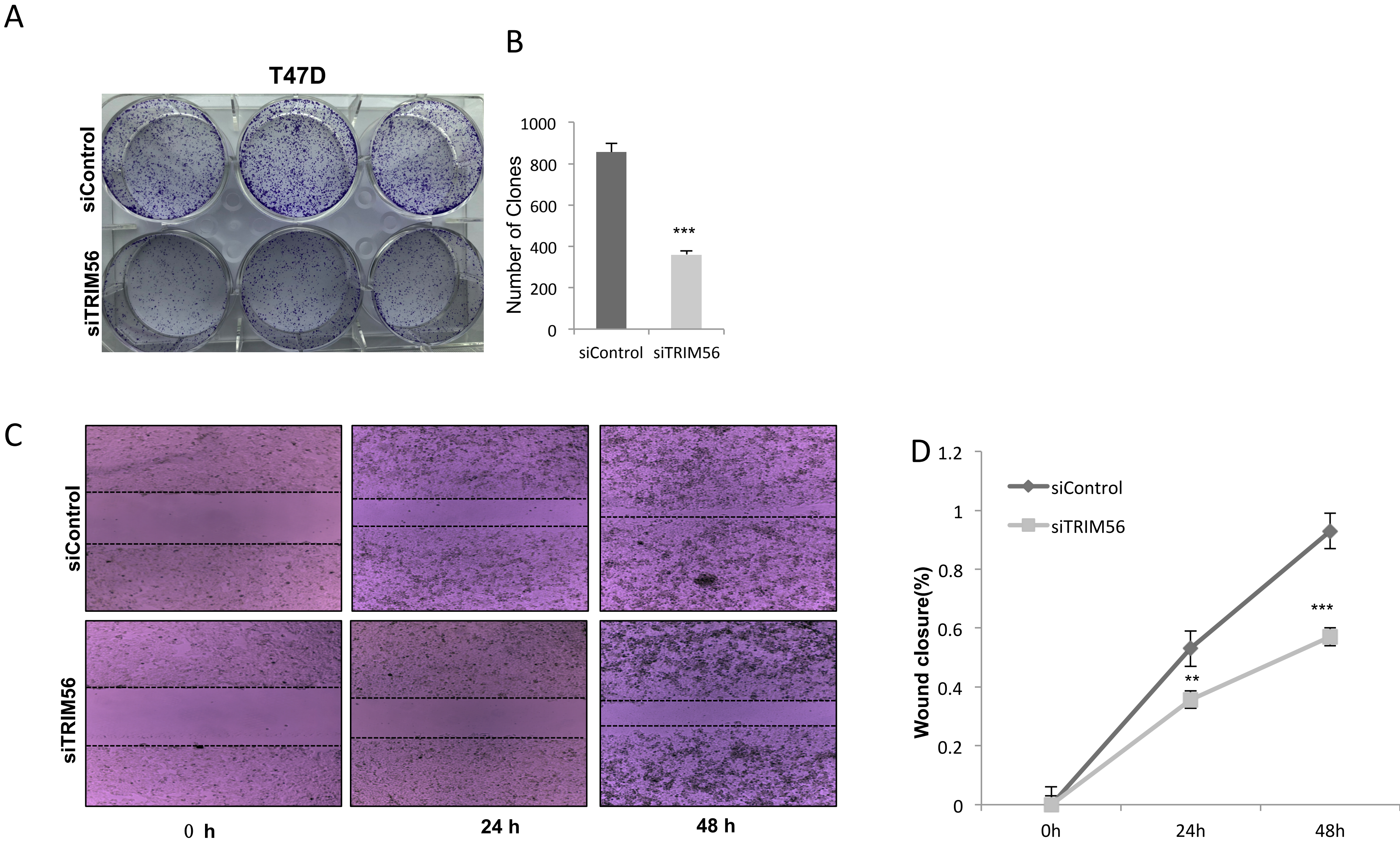

Supplementary figure 4

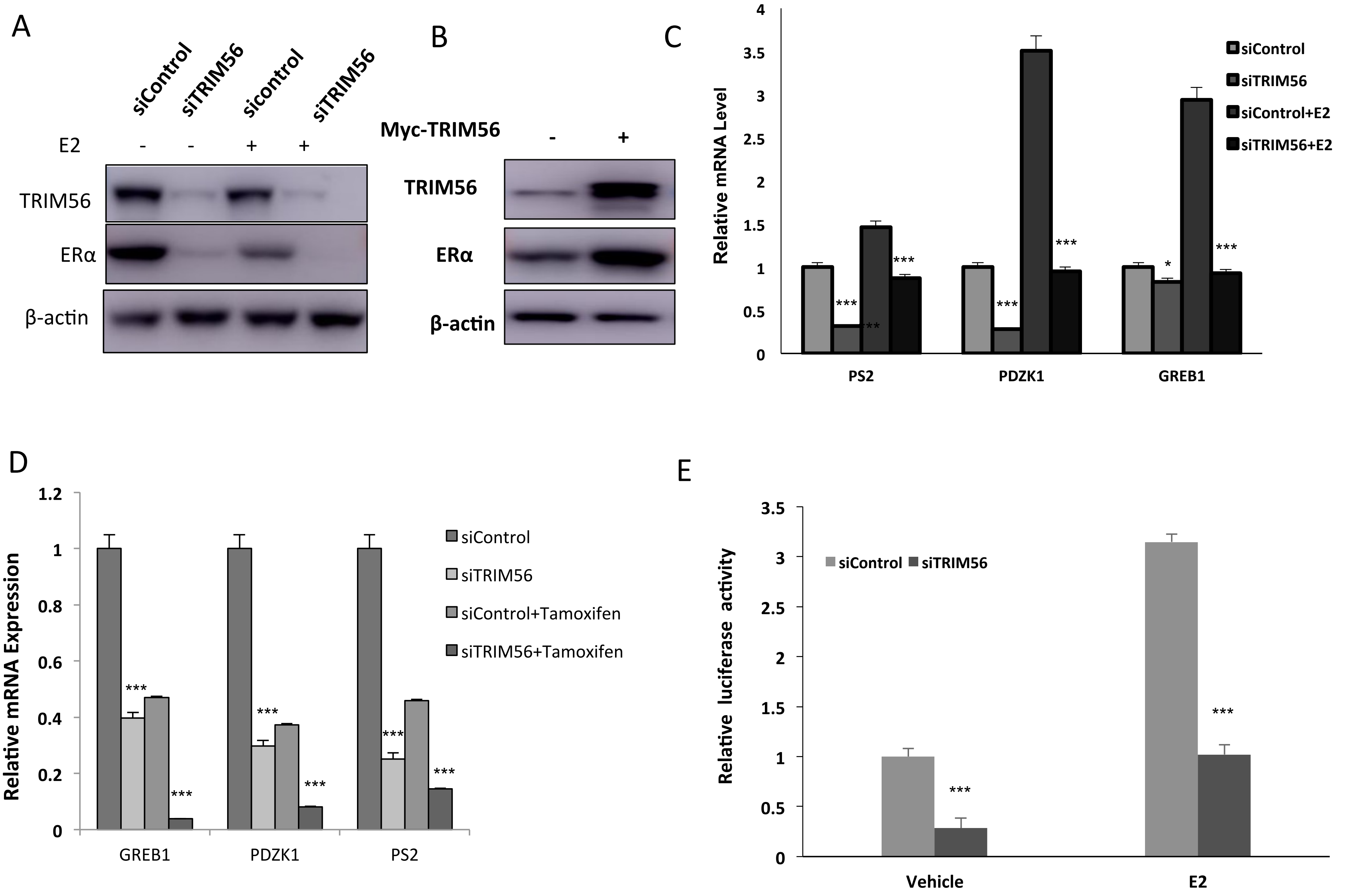

Supplementary figure 5

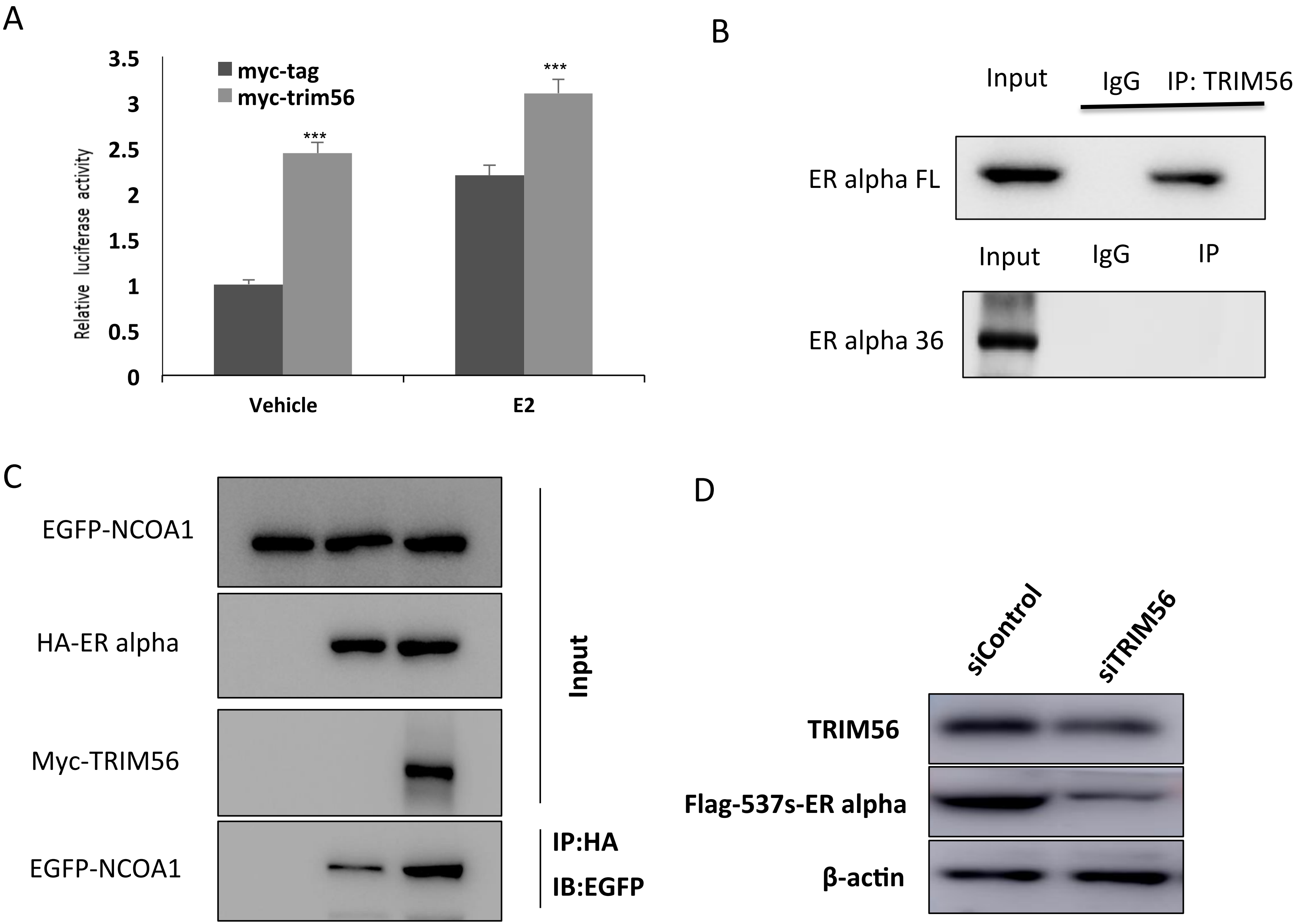

Supplementary figure 6

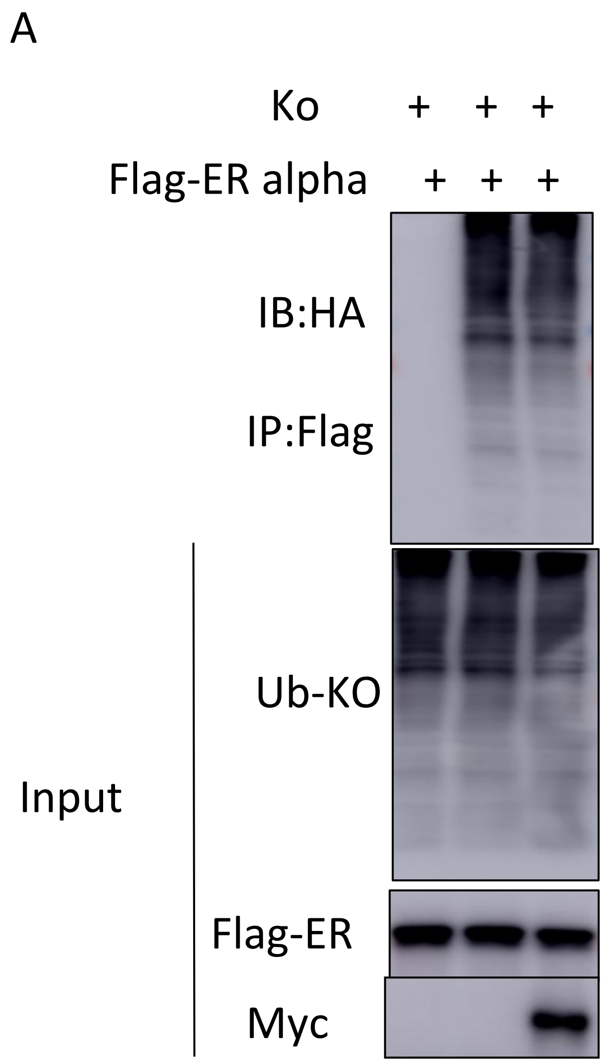

Supplement: Supplementary file 2 — Supplementary Figures [file 41389_2019_139_MOESM2_ESM.pdf]
