# Supplementary material for: Regulation of estrogen signaling and breast cancer proliferation by an ubiquitin ligase TRIM56
Source: Oncogenesis. 2019 Apr 18;8(5):30. doi: 10.1038/s41389-019-0139-x (PMC6473003; doi:10.1038/s41389-019-0139-x)
Supplement: Supplementary file 3 — Supplementary table [file 41389_2019_139_MOESM3_ESM.pdf]

Supplementary table 1

gene_short_name	sicontrol_1	sicontrol_2	sicontrol_3	siTRIM56_1	siTRIM56_2	siTRIM56_3
ESR1	63.52726301	63.6379926	63.70861705	61.93742547	61.13834923	61.58497623
IL20	63.84186415	63.36177012	63.52916175	57.32690605	57.75438376	57.46467231
CXCL12	57.07213241	57.37625166	57.0393915	52.84855454	52.58146824	52.52592272
N4BP3	57.33399437	57.4000975	57.26740045	54.82493982	54.85859479	55.09926246
CELSR2	59.36055592	59.35699132	59.15357876	50.39759789	51.07013244	51.51477579
XBP1	82.25227425	82.08782798	81.92651816	79.31476822	79.3044429	79.11489003
ZNF703	59.98424178	59.83521321	59.88949528	57.11111624	56.77358945	56.71502561
UPF1	60.23842421	60.28167009	60.20426054	58.80894071	58.99290844	58.98753649
MTDH	69.28433738	69.60807842	69.27258949	65.7825294	66.01666867	66.27937584
PDZK1	53.23253501	52.75544132	52.81670496	47.21734599	47.06038029	47.93024062
H2AFY	71.68684792	71.57641959	71.74922592	65.21854277	66.20035454	66.18197377
ABCA3	57.5825658	58.12983234	57.97876134	50.73227417	50.97064784	52.06370563
RCL1	59.48098984	59.28777943	58.92095595	56.67150654	56.40599357	56.60602971
INHBB	57.52820485	57.20632995	57.18085174	54.79152453	54.79899388	55.15535788
MSI2	67.6432842	67.49713059	67.37064274	65.22619083	64.72737816	64.7519316
TFF1	86.61944396	85.86732908	85.74447602	81.60291051	82.00945087	81.94407448
ACSS1	50.103648	49.95230431	49.9866649	44.25175552	44.59967208	45.52351992
SMCR7L	56.83831239	57.27803937	57.02555633	55.27400813	55.20578643	55.31619262
ELOVL5	66.75029951	66.62499441	66.59336841	64.45170772	64.82239676	64.92637773
PBX1	62.36684604	62.22615019	61.86227965	60.14384992	59.85750441	60.01472195
SYPL1	72.63713026	72.67125469	72.38285201	69.76045484	70.36376823	70.13683497
HIVEP3	55.70360359	55.16569506	54.89597423	51.32974255	50.95632695	51.72302253
MYC	71.64017273	71.81569858	71.80435152	69.6720136	69.00481948	69.36971032
BCL2	63.34911217	62.40653561	62.40252452	58.39481585	57.67270471	58.36420982
IL24	55.60880716	54.72791001	55.17163666	51.54851	52.0551808	51.46317882
TMEM164	57.10241941	57.35909819	57.94624587	52.99023382	53.50467699	53.92450148
SARS	75.64303201	76.02765406	75.66059367	74.426798	74.30648353	74.36031842
HS6ST1	58.78868975	58.79864908	58.62734339	57.50644905	57.802295	57.58156737
FRS2	56.29284707	56.51196381	56.4509362	55.2204924	55.10934429	54.84169465
UBE2T	73.14247375	73.14334944	73.22131011	72.27417903	72.48167799	72.23736179
BAMBI	71.48190529	71.60561903	71.7017984	67.52416035	68.22851862	68.73839094
ZNF318	51.11890583	51.13194705	50.81560398	49.89783701	49.6389872	49.83118839
PDLIM3	50.44972742	50.14615942	50.35367661	48.06662917	47.24546894	47.94893356
RNF144B	46.63651148	46.94037071	46.43256202	44.91927894	45.07828271	44.67213996
SMOC2	47.01467668	46.97079608	47.32996978	44.8687773	44.79393511	45.40228983
PKIB	74.20109953	74.01350875	73.82279681	71.79746733	71.50613956	70.90727833
JAK2	49.29647071	49.62817016	49.27297115	45.4439442	46.61253779	46.20974554
PDCD4	74.97788705	75.31561957	75.31867418	73.43080289	73.85328918	73.7156507
MYB	61.06696307	60.89946292	61.00180042	58.77163227	59.26697428	59.4640621
GREB1	67.23571388	67.00454437	66.83802888	64.05912124	64.23955122	64.9811843
TFAP2C	65.30579842	65.6663461	65.79047304	64.07334527	63.54591223	63.75737522
ITPRIP	47.99619225	47.79295746	47.84879975	45.99861046	45.89682261	46.53390016
LAPTM4B	73.71850665	73.64901963	73.57981016	73.0033638	72.93205295	73.14883821
JKAMP	65.18841153	64.82209302	64.29132564	62.53393074	62.57903736	62.70569764
PMAIP1	53.50262808	53.64950327	52.87304936	51.10657705	50.7892539	50.36210419
RCC2	67.99869265	68.37913027	68.16332087	67.21406629	67.27346954	67.31707414
TPD52L1	74.47620288	74.58233149	74.42574358	73.8979008	73.87640796	73.66506785
MED13L	67.45585604	67.34930583	66.97005021	64.29631581	64.41117005	65.25098985
IRX5	56.40970813	56.69261945	56.75359944	53.89639947	54.84089727	54.20130284
OLFM1	61.75845541	61.42719589	62.12470351	57.93991305	58.7584645	59.17288404
EGR3	52.55565544	53.31462433	53.01857948	50.92025545	50.6440869	51.19152709
MSX2	54.51445878	53.89661608	54.612822	52.07146178	52.35133974	52.62562678
SMIM15	65.64251781	64.61895136	65.68638437	62.82313745	62.54892439	62.79428539
